# Low back pain education and short term quality of life: a randomized trial

**DOI:** 10.1186/1471-2474-8-21

**Published:** 2007-02-28

**Authors:** Sedigheh Sadat Tavafian, Ahmadreza Jamshidi, Kazem Mohammad, Ali Montazeri

**Affiliations:** 1 Iranian Institute for Health Sciences Research, Tehran, Iran; 2 Rheumatology Research Center, Tehran University of Medical Sciences, Tehran, Iran; 3 School of Public Health, Tehran University of Medical Sciences, Tehran, Iran; 4 Public Health and Health Policy, Division of Community-Based Sciences, University of Glasgow, Glasgow, UK

## Abstract

**Background:**

Different interventions can reduce the burden of the chronic low back pain. One example is the use of a 'Back School Programme'. This is a brief therapy that uses a health education method to empower participants through a procedure of assessment, education and skill development. This study aimed to evaluate to what extent the programme could improve quality of life in those who suffer from the condition.

**Methods:**

This was a randomized controlled trial. One-hundred and two female patients with low back pain (n = 102) were randomly allocated into two groups, matched in terms of age, weight, education, socioeconomic status, occupation and some aspects of risk behavior. Group 1 (back school group, n = 50) but not group 2 (clinic group, n = 52) received the 'Back School Programme'. Then quality of life using the Short Form Health Survey (SF-36) was assessed at two time points: at baseline and at three months follow-up. The findings were compared both within and between two groups.

**Results:**

The 'Back School Programme' was effective in improving patients' quality of life; significant differences were found on all eight subscales of the SF-36 for group 1. In the clinic group (group 2), improvement was observed on three scales (bodily pain, vitality and mental health) but these improvements were less than in group 1. The mean improvement over all eight subscales of the SF-36 was significantly better for the 'Back School Programme' group.

**Conclusion:**

The 'Back School Programme' is an effective intervention and might improve the quality of life over a period of 3 months in patients who experience chronic low back pain.

## Background

Chronic low back pain is a common health problem in many countries. Individuals suffering from chronic low back pain experience major physical, social, mental, and occupational disruptions [1]. It is argued that the impact of low back pain includes: loss of physical function; deterioration of general health and reconditioning (loss of muscle tone and weight gain); constant or episodic pain or increase in the level of pain; loss of social functioning manifested as decreased participation in social and leisure activities, family stress, or loss of group and community relatedness (often associated with decreased income and/or job loss); and disruption of psychological functioning manifested through insomnia, irritability, anxiety, depression and somatic complaints [2,3].

It has been shown that different interventions can reduce the burden of the disease. One example is the use of a 'Back School Programme'. This is a brief outpatient programme that uses a health education approach to empower participants through a process of assessment, education and skill building, leading to improved quality of life. The predominant interventions used in the 'Back School Programme' are physical training, physical therapy and exercises. It has been shown that the programme reduces back pain, decreases the time lost from work and improves patient functioning [4]. However, little information exists on the Back School Programme with quality of life as the outcome measure [3]. There is a consensus that clinical trials designed to assess the efficacy and effectiveness of treatments for chronic pain should consider outcomes in six core domains: pain, physical functioning, emotional functioning, patient global ratings of satisfaction, negative health states, and adverse events, and patient disposition [5]. The purpose of this study was to examine whether the 'Back School Programme' could improve patients' health-related quality of life.

## Methods

### Study design and data collection

This was a blind randomized controlled trial with a 3-month follow-up undertaken in the Rheumatology Research Center of Tehran University of Medical Sciences, Tehran, Iran from July to September 2003. Eligible participants were adult women recruited from outpatient rheumatology clinics. The selection criteria were: age 18 years and over, suffering from chronic back pain (persisting for 90 days or more), and having a telephone number for regular contact with a responsible caregiver. Patients were excluded from the study if they had had back surgery within the two years prior to the initial observation, or if the complaint was restricted to the sacroiliac joint or the cervical or thoracic regions, or if there was congenital spine disease. Patients with a low back complaint that had persisted less than 90 days were also excluded. Physicians confirmed the inclusion and exclusion criteria through a complete and exact clinical assessment before the participants were enrolled in the study. All patients had some kind of chronic low back pain and were examined and treated by only one rheumatologist throughout the study. To participate, patients had to be willing to comply with the entire study protocol. Therefore, the procedures were described, the purposes of the study were explained and written consent was sought before any part of the study procedure was administered or any medication or intervention was dispensed. The ethics committee of Tehran University of Medical Sciences approved the study.

Randomization was scheduled every week after preparation of the list of patients. The patients (n = 102) were randomly assigned at the outset to a clinic group (52 participants), who received only medication under the supervision of a leading physician, and a 'Back School Programme' group (50 participants), who received both medication and the 'Back School Programme' as intervention. The medication for both groups was the same (Acetaminophen, NSAID, and Chlordiazepoxide) and the treatment allocation was not concealed. In addition cointerventions were avoided for both group and patients were not blinded to the intervention. Of the 52 subjects randomly allocated to the clinic group (group 2), 5 dropped out during the study: one withdrew consent and four were lost to follow-up. Thus, a total of 47 subjects who met the study criteria and completed the survey were finally included in group 2. Of the 50 patients randomly allocated to the Back School Programme (group 1), six were excluded from the study during the run-in, two withdrew consent, and four failed to comply with the programme. Therefore, 44 patients in group 1 completed the entire 3-month study. It should be emphasized that none of the dropouts was due to the intervention.

Data from both groups were collected at admission and after the 3-month treatment period. Both groups received an initial physician evaluation, subsequent treatment as determined by physicians, and follow-up physician visits. The Back School Programme patients (group 1) received one additional interdisciplinary evaluation and a four-day interdisciplinary educational intervention than the clinic group (group 2). Also, the group 1 patients were reassessed by a physiotherapist at the end of the first week and given weekly follow-ups by a health educator to encourage them to comply the intervention.

### Study measures

1. Basic demographic data: This questionnaire covered age, weight, socioeconomic status and some risk behaviors regarding low back pain and was completed during interviews with the patients.

2. The Short Form Health Survey (SF-36): This is a well known general quality of life questionnaire that measures health-related functioning in eight subscales: physical functioning (PF), role limitations due to physical problems (RP), bodily pain (BP), vitality (VT), general health perceptions (GH), social functioning (SF), role limitations due to emotional problems (RE) and mental health (MH). The SF-36 reports the patients' perceived quality of life by scores ranging from zero to 100, where 100 is the best and zero is the worst score [6]. We used the Iranian version of the SF-36 questionnaire. The validity and reliability of the Iranian translation of the SF-36 is well documented [7].

### Study intervention

The 'Back School Programme' is a four-day, five-session, multidimensional and interdisciplinary educational regime designed to assess each patient's physical condition, personal characteristics, lifestyle and subsequent ability to cope. The goal is to assist patients to attain the highest levels of functioning possible in view of their diseases and treatments. The programme utilizes an empowerment approach, providing a combination of knowledge, skills, and heightened self-awareness regarding values and needs, so that patients can define and achieve their own goals [8]. Additionally, each patient is an active member of the team, contributing communication-building and problem-solving skills. Therefore, the knowledge, awareness, perceptions, skills and needs of the patients were initially assessed by a focus group discussion and then the educational programme was designed on the basis of modern back school items and these assessments. The members of the educational team had the following responsibilities. A PhD-level educator assessed the knowledge, perceptions and beliefs of the patients concerning health, the contributions of non-healthy behaviors to low back pain and approaches to changing non-healthy behavior, and motivated the patients to adopt more healthy behavior. A clinical psychologist conducted psychological evaluations and diagnoses and facilitated the focus on individual coping skills, anger management and relaxation in the patient group. A rheumatologist obtained health histories and conducted the back school classes, which included the anatomy and physiology of the spine, so that each patient could understand how a normal, healthy spine functions and how proper movements can protect it and help in pain management. The patients were also instructed in the natural history of spinal conditions, lifestyle factors that accelerate the chronic low back pain process, and techniques for preventing further injury. The rheumatologist helped the patients to understand the diagnostic tools utilized by physicians, and described the treatment options and associated risks. A physical therapist conducted a comprehensive evaluation and provided instruction in lumbar stabilization, body mechanics and prevention techniques. He also performed a conditioning and aerobic capacity evaluation and developed a weight-bearing exercise and optimal aerobic fitness programme for each patient. In addition, he conducted classes to improve the knowledge and skills of patients in respect of muscle stretching and strengthening and relaxing exercises for the back, abdomen and thighs.

### Study outcome

The principal outcome measure was quality of life in the two groups. The mean increase in quality of life score above the baseline was used as the main outcome measure of the patients' responses to the intervention.

### Statistical analysis

The study used the intention-to-treat analysis. For categorical and continuous data comparison was made using the chi-square (Fisher's exact test where necessary) and t-test respectively. Since most scores on the SF-36 were not normally distributed, the Mann-Whitney U-test was performed to compare quality of life scores between two groups at baseline and at follow-up. In addition non-parametric paired test (Wilcoxon Signed Ranks test) was performed to compare the SF-36 scores for each group at baseline and follow-up assessments.

## Results

The 102 patients were assessed and randomly allocated to two groups: group 1 or Back School Programme (50 patients), and group 2 or clinic group (52 patients). Table 1 shows the basic demographic characteristics and some risk behaviors of the patients. The results showed no statistically significant differences between the two groups in terms of these baseline data (all P values > 0.05).

The means scores of the eight subscales of the patients at the commencement of the trial are shown in Table 2. The Mann-Whitney test showed no baseline differences between the two groups with regard to these subscales.

The improvements in group 1 over three months were strongly significant on all subscales (P < 0.001). However, in group 2, significant improvements were revealed only on three subscales: bodily pain (P = 0.001), vitality (P = 0.02) and mental health (P = 0.04). Although these were significant they were much less than the corresponding improvements in group 1. The results are show in Table 3.

## Discussion

This randomized trial showed that the back programme education patients improved significantly on all quality of life subscales. In the clinic group, although improvements were seen on all subscales, they were much less than those in the back programme group and significant only on the bodily pain, vitality and mental health subscales. The ability of the back programme to effect significant improvements on all quality of life subscales for its patients, in contrast to the clinic alone, points to the likely strength of the programme in these areas. In contrast to previous studies [3] we showed that the 'Back School Programme' not only was effective in improving patients' physical functioning but also had effect on mental component of patients' quality of life. A recent study on the topic has found that the health related quality of life of patients with low back pain depended on functional status and psychological factors more than simple physical impairment [8]. Thus, in this respect it seems that the 'Back School Programme' is a very relevant regimen to improve both patients' physical and psychological status.

A strong point of the back programme was the highly significant improvement on the bodily pain, vitality and mental health subscales scores. These were much more marked in the back programme group than in the clinic group, and analysis showed that the mean differences in improvements between the two groups were significant. This comparison, although speculative, helps to forestall possible objections to the pre-post design used here.

The improvement in all quality of life scales might be related to two factors: (i) the reduction of bodily pain which eased the performance of daily activities, and (ii) diminishing the risk of disability due to leaning to have a more healthy body mechanics. This is an important finding for two reasons. The most obvious is that one of the goals of the back programme is to restore the participants to the highest possible level of functioning. Significantly increasing functioning and lowering disability risk are key elements in attaining this goal. The second is that most of the back programme patients had a chronic disabling lumbar condition. Diminishing the risk of disability indicates that the back programme had a positive impact on this complex and resistant phenomenon.

Although the mental health and vitality scores of the clinic group improved significantly over the three month period owing to antidepressant and analgesic medication, the improvements in these scores were more significant in group 1. This finding is related to one of the most important objectives of the back programme: to assess the patients and offer psychosocial self-help skills and referrals over the brief three-month period. The ability of the 'Back School Programme' to implement the recommendations of psychotherapists in respect of stress control and problem solving, and to elicit further recommendations concerning psychiatric evaluations and psychotherapy visits during weekly follow-ups, improved the mental status of patients in this group. Other factors in the back programme group such as exercise therapy might also have improved the mental health status of the patients more than in the clinic group. There is evidence that exercise training could improve functional ability and quality of life in patients with low back pain [9]. However, it is argued that it is impossible to define a generic set of predictors of outcome of back school for patients with chronic low back pain [10].

All patients in present study were women. Thus the results might not be generalized to all patients with low back pain. In addition one might question about a high proportion of housewives in the study (88% in the intervention group and 75% in the control group) and argue that since these women were housewives and were less active (physically and socially) therefore the results based on the chosen outcome (quality of life as measured by the SF-36) are biased and could not be related to the 'Back School Programme'. In Iran being a housewife involves a considerable amount of physical and social activities and thus we believe the high proportion of housewives in the study did not affect the results. However, we recommend in future studies include a more heterogeneous sample of low back patients and also assess perceived ability to work as an important outcome measure. Unfortunately the current study did not measure this and thus we are unable to comment on such a significant outcome. Future studies also should include a non-specific activity of the control group of a same duration as the intervention group. A further limitation is the rather short disease duration of 9 months, compared with typical patients suffering from non-specific chronic low back pain since several years.

## Conclusion

The findings from this randomized trial suggest that the 'Back School Programme' is an effective intervention and could play an important role in improving quality of life in patients who suffer from the chronic low back pain.

## Competing interests

The author(s) declare that they have no competing interests.

## Authors' contributions

SST was the main investigator, carried out the study, contributed to the analysis and wrote the first draft. AJ contributed to the study design, recruitment of patients and clinical aspect of the study. KM contributed to the study design and statistical analysis. AM carried out the analysis and wrote the final draft. All authors read and approved the final manuscript.

## Pre-publication history

The pre-publication history for this paper can be accessed here:



## Figures and Tables

**Table 1:** The characteristics of the Back School Programme and clinic groups at baseline

	**Back School group (n = 50)**		**Clinic group (n = 52)**		**P**
	**Mean (SD)**	**No. (%)**	**Mean (SD)**	**No. (%)**	

**Age (year)**	42.9 (10.7)		44.7 (10.8)		0.4
**Weight (Kg)**	68.7 (10.4)		69.1 (12.5)		0.8
**Pain duration (months)**	8.90 (3.2)		9.24 (3.2)		0.5
**Sciatica**					0.7
Yes		11 (22)		10 (19)	
No		39 (78)		42 (81)	
**Education**					0.1
Primary		10 (20.0)		15 (28.8)	
Secondary		15 (30.0)		11 (21.2)	
Diploma		19 (38.0)		18 (34.6)	
College/higher		6 (12.0)		8(15.4)	
**Occupation**					0.1
Employed		6 (12.0)		13 (25.0)	
Housewife		44 (88.0)		39 (75.0)	
**Household income**					0.3
High		24 (48.0)		20 (38.5)	
Moderate		23 (46.0)		25 (48.1)	
Low		3 (6.0)		7 (13.5)	
**Smoking**					0.3
Non-smoker		47 (94.0)		51 (98.1)	
Smoker		3 (6.0)		1(1.9)	
**Do exercise**					0.6
Always (every day)		8 (16.0)		5 (9.6)	
Occasionally (2 to 3 times in a week)		22 (44.0)		26 (50.0)	
Never		20 (40.0)		21 (40.4)	
Using proper chair*					0.5
Yes		22 (44.0)		26 (50.0)	
No		28 (56.0)		26 (50.0)	
Using proper shoes*					0.8
Yes		34 (68.0)		34 (65.4)	
No		16 (32.0)		18 (34.6)	
Using proper bed*					0.6
Yes		34 (68.0)		38 (73.1)	
No		16 (32.0)		14 (26.9)	

* Proper chair: chairs with straight backs or low back support and also knee support to keep the knees a little higher than the hips. Proper shoes: shoes with low heels (1 inch or lower). Proper bed: medium-firm mattresses that provide support while allowing for natural curves and alignment of the spine.

**Table 2:** Comparison of the SF-36 scores between the two groups at baseline (a higher value indicates a better condition)

	**Back School group (n = 50) Mean (SD)**	**Clinic group (n = 52) Mean (SD)**	P*
**Physical Functioning**	52.9 (24.9)	52.5 (20.2)	0.90
**Role Physical**	27.5 (26.8)	31.7 (35.0)	0.89
**Bodily Pain**	41.8 (19.6)	42.6 (25.3)	0.61
**General Health**	40.7 (24.6)	41.7 (22.2)	0.92
**Mental Health**	49.8 (28.2)	47.8 (23.5)	0.71
**Role Emotional**	34.0 (41.1)	32.7 (40.4)	0.89
**Vitality**	46.7 (23.9)	48.9 (21.6)	0.62
**Social Functioning**	59.2 (29.9)	62.5 (29.8)	0.61

* Mann-Whitney test.

**Table 3:** Comparison of the SF-36 scores in Back School and clinic groups at baseline and at follow-up assessments (a higher value indicates a better condition)

	**Back School group (n = 44)**		P**	**Clinic group (n = 47)**		P**
Scales*	**Baseline Mean (SD)**	**Follow-up Mean (SD)**		**Baseline Mean (SD)**	**Follow-up Mean (SD)**	

**PF**	55.5 (24.0)	79.3 (18.6)	< 0.001	53.4 (20.8)	54.4 (27.0)	0.58
**RP**	31.2 (26.4)	78.9 (28.5)	< 0.001	32.9 (35.7)	40.9 (36.6)	0.11
**BP**	43.4 (19.6)	71.5 (16.2)	< 0.001	43.5 (25.8)	56.6 (30.0)	0.001
**GH**	43.9 (23.1)	61.6 (22.7)	< 0.001	42.2 (22.4)	47.3 (26.1)	0.51
**MH**	52.7 (28.0)	74.0 (22.8)	< 0.001	48.8 (22.9)	54.3 (26.6)	0.04
**RE**	35.6 (42.0)	72.8 (40.6)	< 0.001	32.6 (40.4)	34.0 (42.4)	0.79
**VT**	48.7 (23.4)	73.2 (22.0)	< 0.001	48.6 (21.4)	56.8 (25.6)	0.02
**SF**	62.5 (28.2)	87.7 (21.6)	< 0.001	64.0 (29.3)	69.1 (32.7)	0.15

* PF = Physical functioning, RP = Role physical, BP = Bodily pain, General health, MH = Mental health, RE = Role emotional, VT = Vitality, SF = Social functioning.

** Wilcoxon Signed Ranks test.

## References

[B1] MacDonald MJ, Sorock GS, Volinn E, Hashemi L, Clancy EA, Webster B (1997). A descriptive study of recurrent low back pain claims. J Occup Environ Med.

[B2] Patrick DL, Erickson P (1993). Health status and health policy: quality of Life in health care evaluation and resource allocation.

[B3] Claiborne N, Vandenburgh H, Krause TM, Leung P (2002). Measuring quality of life changes in individuals with chronic low back conditions: a back education programme evaluation. Evaluation and Programme Planning.

[B4] Lonn JH, Glomsrod B, Soukup MG, Bo K, Larsen S (1999). Active back school: prophylactic management for low back pain: a randomized controlled 1-year follow-up study. Spine.

[B5] Turk DC, Dworkin RH (2004). What should be the core outcomes in chronic pain clinical trials?. Arthritis Res Ther.

[B6] Ware JE, Snow KK, Kosinski M, Gandek B (1993). SF-36 Health survey-Manual and interpretation guide.

[B7] Montazeri A, Goshtasebi A, Vahdaninia M, Gandek B (2005). The Short Form Health Survey (SF-36): translation and validation study of the Iranian version. Qual Life Res.

[B8] Horng YS, Hwang YH, Wu HC, Liang HW, Jang Y, Twu FC, Wang JD (2005). Predicting health-related quality of life in patients with low back pain. Spine.

[B9] Shaughnessy M, Caulfield B (2004). A pilot study to investigate the effect of lumbar stabilization exercise training on functional ability and quality of life in patients with chronic low back pain. Int J Rehabil Res.

[B10] Hulst M, Vollenbroek-Hutten M, Ijzerman M (2005). A systematic review of sociodemographic, physical, and psychological predictors of multidisciplinary rehabilitation or back school treatment outcome in patients with chronic low back pain. Spine.

